# Calciprotein Particle Synthesis Strategy Determines In Vitro Calcification Potential

**DOI:** 10.1007/s00223-022-01036-1

**Published:** 2022-11-03

**Authors:** Lara W. Zeper, Edward R. Smith, Anique D. ter Braake, Paul T. Tinnemans, Jeroen H. F. de Baaij, Joost G. J. Hoenderop

**Affiliations:** 1 Department of Physiology, Radboud Institute for Molecular Life Sciences, Radboud University Medical Center, P.O. Box 9101, 6500HB Nijmegen, The Netherlands; 2 Department of Nephrology, The Royal Melbourne Hospital, Parkville, VIC Australia; 3 Department of Nephrology, University of Melbourne, Parkville, VIC Australia; 4 Institute for Molecules and Materials, Radboud University, Nijmegen, The Netherlands

**Keywords:** CPP, Vascular calcification, Calcium content, Chronic kidney disease

## Abstract

Circulating calciprotein particles (CPP), colloids of calcium, phosphate and proteins, were identified as potential drivers of the calcification process in chronic kidney disease. The present study compared CPP produced using different protocols with respect to particle morphology, composition, particle number and in vitro calcification potency. CPP were synthesized with 4.4 mM (CPP-A and B) or 6 mM (CPP-C and D) phosphate and 2.8 mM (CPP-A and B) or 10 mM (CPP-C and D) calcium, with either bovine fetuin-A (CPP-C) or fetal bovine serum (CPP-A, B and D) as a source of protein, and incubated for 7 (CPP-A2) or 14 days (CPP-B2), 12 h (CPP-C2, D2 and B1) or 30 min (CPP-D1). Particle number was determined with nanoparticle tracking and calcium content was measured in CPP preparations and to determine human vascular smooth muscle cell (hVSMC) calcification. Morphologically, CPP-C2 were the largest. Particle number did not correspond to the calcium content of CPP. Both methods of quantification resulted in variable potencies of CPP2 to calcify VSMC, with CPP-B2 as most stable inducer of hVSMC calcification. In contrast, CPP-B1 and D1 were unable to induce calcification of hVSMC, and endogenous CPP derived from pooled serum of dialysis patients were only able to calcify hVSMC to a small extent compared to CPP2.

CPP synthesized using different protocols appear morphologically similar, but in vitro calcification potency is dependent on composition and how the CPP are quantified. Synthetic CPP are not comparable to endogenous CPP in terms of the calcification propensity.

**Supplementary Information:**

The online version contains supplementary material available at 10.1007/s00223-022-01036-1.

## Introduction

Cardiovascular complications are the main cause of death in patients with chronic kidney disease (CKD) [1]. As a result of renal failure, serum phosphate (P_i_) concentrations are elevated in CKD patients. P_i_ is a strong promotor of vascular calcification and is associated with an increased risk for cardiovascular mortality [2–4].

Recently, several groups have demonstrated that increased serum P_i_ levels lead to the formation of calciprotein particles (CPP). CPP consist of P_i_, calcium (Ca^2+^) and serum proteins such as the liver-derived mineral-binding protein, fetuin-A [5]. In the CKD-associated calcification milieu, amorphous primary CPP (CPP1) transform spontaneously into crystalline, hydroxyapatite-containing secondary CPP (CPP2). The transition from CPP1 to CPP2 is inhibited by serum components such as fetuin-A, albumin and magnesium [6]. Circulating CPP appear elevated in end-stage kidney disease patients compared to adults without renal impairment, with a greater preponderance of CPP2 [7–10]. The presence of CPP in CKD patients is associated with increased aortic stiffness, coronary artery calcification, and risk of death [7, 11–13]. CPP2 have been shown to directly induce vascular smooth muscle cells (VSMC) calcification in vitro [14–16]. Therefore, CPP2 are considered important drivers of vascular calcification and mediators of phosphate toxicity [17].

To further study the role of CPP in vascular calcification, several research groups have developed protocols to synthesize CPP1 and CPP2 in vitro [5, 6, 8–10, 13, 14, 18–31]. In these studies, supraphysiologic concentrations of Ca^2+^ and P_i_ up to 10 mmol/L are incubated with serum protein(s) to generate CPP. Different protein sources and variable concentrations of P_i_ and Ca^2+^ are used, depending on the protocol. Currently, there is no consensus on how to produce CPP1 or CPP2 synthetically. Therefore, the comparability and reproducibility of the results described in independent studies using synthetic CPP is currently greatly hampered.

This study aimed to investigate the effect of different CPP synthesis strategies on (1) CPP characteristics with respect to their morphology, composition, and quantity, and (2) their calcification potential in vitro. Additionally, characteristics of CPP1 and CPP2 were compared to endogenous CPP isolated from the serum of CKD patients.

## Methods

### Protocol Selection

To collect the most frequently used protocols for synthesizing CPP2 a PubMed search was conducted in March 2019: “calciprotein particles” [All Fields] OR “calciprotein particle” [All Fields] NOT “review” [pt]. Potential articles were excluded when studies lacked a clear description in the method section of CPP2 synthesis consisting of a protein source and specified the source and concentration of P_i_ and Ca^2+^. All included articles are listed in Table 1. To facilitate comparisons between the protocols, NaH_2_PO_4_ was used as P_i_ source in all protocols.

**Table 1:** Overview of all included articles describing CPP formation

Author	Protein source	Concentration P_i_ (final)	Type of P_i_	Concentration CaCl_2_ (final)	Incubation time	Incubation temperature	Solution of incubation	Solution of dissolving
*Aghagolzadeh et al. 2016	10% FBS	4.4 mM	2.14 mM Na_2_HPO_4_, 1.36 mM NaH_2_PO_4_	2.8 mM	7 days	37 °C	Phenol red-free DMEM medium with 1% (v/v) pen/strep and 1% (v/v) l-glutamine	ns
*Aghagolzadeh et al. 2017	10% FBS	4.4 mM	2.14 mM Na_2_HPO_4_, 1.36 mM NaH_2_PO_4_	2.8 mM	7 days	37 °C	Phenol red-free DMEM medium with 1% (v/v) pen/strep and 1% (v/v) l-glutamine	ns
Cai et al. 2015	88 mg/L Human fetuin-A	1.6 mM	Na_2_HPO_4_	ns	6 h	37 °C	Peritoneal dialysis fluid	ns
Cai et al. 2017	Human or fetal calf serum	6 mM	ns	10 mM	Until the opacity changed	37 °C	Saline, Ca^2+^ and P_i_ in HEPES buffered solution	TBS, pH 7.4
Chabriere et al. 2014	1 mg/ml Protein (lysozome, fetuin-A or BSA)	10 mM	NaH_2_PO_4_	10 mM	1–14 days	ns	DMEM medium	ns
*Chen et al. 2018	40% Human serum	6 mM	ns	10 mM	Followed for 6 h	37 °C	NaCl	ns
Heiss et al. 2003	200 nM Bovine fetuin-A	1.8 mM or 3 mM	KH_2_PO_4_ or Na_2_HPO_4_	2.5 mM or 5 mM	1.5–30 h	22 °C or 37 °C	50 mM Tris–HCl, pH 7.4	ns
Heiss et al. 2007	2.5 mg/ml Bovine fetuin-A	6 mM	Na_2_HPO_4_	10 mM	ns	ns	140 mM NaCl, 50 mM Tris–HCl, pH 7.4	ns
Heiss et al. 2008	20 µM Bovine fetuin-A	6 mM	Na_2_HPO_4_	10 mM	> 12 h	RT	140 mM NaCl, 50 mM Tris–HCl, pH 7.4	ns
Heiss et al. 2010	0.5 or 2.5 mg/ml Bovine fetuin-A	6 mM	Na_2_HPO_4_	10 mM	Overnight	RT	140 mM NaCl, 50 mM Tris–HCl, pH 7.4	ns
Herrmann et al. 2012	1 mg/ml Bovine fetuin-A	6 mM	Na_2_HPO_4_	10 mM	1 h	37 °C	140 mM NaCl, 50 mM Tris–HCl, pH 7.4	ns
Ismail et al. 2011	1% FCS or 20 µM Bovine fetuin-A	6 mM	Na_2_HPO_4_	10 mM	Followed over time up to 10 h	RT	50 mM Tris buffer	ns
Kelynack et al. 2016	12.5% FCS	3.5 mM	NaHPO_4_	5 mM	12 h	RT	TBS, pH 7.4	TBS
*Köppert et al. 2018	10% FCS	4.4 mM	ns	2.8 mM	7 days	37 °C	DMEM medium	Saline
*Köppert et al. 2018	1 mg/ml Bovine fetuin-A	6 mM	ns	10 mM	12 h	37 °C	140 mM NaCl, pH 7.4	Saline
Miura et al. 2018	2.0 g/ml BSA and 0.5 mg/ml bovine fetuin-A	2 mM	‘phosphate buffer’ pH 7.4	3 mM	24 h	37 °C	DMEM medium	ns
*Pasch et al. 2012	40% Human or mouse serum	6 mM	19.44 mM Na_2_HPO_4_, 4.56 mM NaH_2_PO_4_	10 mM	ns	37 °C	140 mM NaCl + 100 mM HEPES, pH 7.4	ns
Rochette et al. 2009	Bovine fetuin-A (1, 5 or 15 µM)	6 mM	Na_3_PO_4_	10 mM	ns	37 °C	50 mM Tris–HCl, pH 7.4	ns
Smith et al. 2012	0.7 mg/ml Human fetuin-A	6 mM	Na_2_HPO_4_	10 mM	12 h	24 °C	50 mM Tris–HCl, pH 7.4	ns
*Smith et al. 2013	1 mg/ml Human fetuin-A	6 mM	NaH_2_PO_4_	10 mM	24 h	37 °C	140 mM NaCl, 50 mM Tris–HCl, pH 7.4	Washed in TBS and resuspended in “pre-warmed buffer”
*Smith et al. 2017	40% Human serum	6 mM	19.44 mM Na_2_HPO_4_, 4.56 mM NaH_2_PO_4_	10 mM	12 h	37 °C	140 mM NaCl, 25 mM Tris buffer, pH 7.4	TBS
*Smith et al. 2018	40% Human serum	6 mM	19.44 mM Na_2_HPO_4_, 4.56 mM NaH_2_PO_4_	10 mM	12 h	37 °C	140 mM NaCl, 25 mM Tris buffer, pH 7.4	TBS
Wu et al 2015	5% FBS	10 mM	Na_2_HPO_4_	10 mM	Overnight—1 month	22 °C	DMEM medium	20 mM HEPES, 1 mM CaCl_2_, 2 mM Na_2_HPO_4_, 150 mM NaCl

*The protocols of these articles were combined to the 4 different protocols

*BSA* bovine serum albumin, *FBS* fetal bovine serum, *FCS* fetal calf serum, *ns* not specified, *Pi* phosphate, *TBS* tris-buffered saline

### Generation and Isolation of Synthetic CPP1 and CPP2

CPP1 and CPP2 were generated according to two and four different protocols, respectively (summarized in Table 2). All CPP mixtures were prepared in open-cap T75 flasks with a final medium volume of 50 mL with gentle swirling between any addition. The CPP mixtures were prepared as follows. First, either 10% v/v FBS (Batch No. S14344S1810, BioWest, GE healthcare, Little Chalfont, UK) (CPP-A/B), 40% v/v FBS (CPP-D) or 1 mg/mL bovine fetuin-A (F2379, Sigma, Saint Louis, Missouri, USA) (CPP-C) was added to pre-warmed incubation solution at 37 °C. For CPP-A/B phenol-red free Dulbecco’s modified eagle medium (DMEM, #21063029, Gibco, Thermo Fisher Scientific, Waltham, Massachusetts, USA), 1 mmol/L sodium pyruvate (Gibco), 0.1 mmol/L non-essential amino acids (GE Healthcare) and antibiotics (either 10 μg/mL ciprofloxacine or 100 U penicillin and 100 μg streptomycin) or M199 (Gibco) supplemented with 2 mmol l-glutamine and antibiotics was used as incubation solution and 140 mmol/L NaCl, 50 mmol/L Tris–HCl set to pH 7.4 for CPP-C/D. Second, P_i_ (NaH_2_PO_4_) was added to a final concentration of 4.4 mmol/L in CPP-A/B and 6 mmol/L in CPP-C/D. Third, a concentrated CaCl_2_ stock was added to reach a final concentration of 2.8 mmol/L in CPP-A/B and 10 mmol/L in CPP-C/D. Finally, the mixtures were swirled gently and incubated for 14 days (CPP-B2), 7 days (CCP-A2), 12 h (CPP-B1, C2 and D2) or 30 min (CPP-D1) in a humidified incubator at 37 °C containing 5% CO_2_ (v/v). To isolate CPP from the mixtures, samples were centrifuged in a high-speed centrifuge for 2 h at least 24,000×*g* at 4 °C and washed in Tris-buffered saline (TBS). Subsequently, CPP were re-centrifuged before resuspending in TBS. Isolated CPP were used immediately for the calcification experiments and the remaining samples were stored at 4 °C or – 80 °C for 14 days.

**Table 2:** Frequently used protocols in literature included in this paper

Protocol name	Protein source	Concentration P_i_ (final)	Type of P_i_	Concentration CaCl_2_ (final)	Incubation time	Incubation temperature	Incubation buffer	Storage solution
CPP-B1	10% FBS	4.4 mM	NaH_2_PO_4_	2.8 mM	12 h	37 °C	Phenol red-free DMEM medium + 1% (v/v) sodium pyruvate and 1% (v/v) NEAA and antibiotics	TBS
CPP-D1	40% FBS	6 mM	NaH_2_PO_4_	10 mM	30 min	37 °C	140 mM NaCl, 50 mM Tris–HCl, pH 7.4	TBS
CPP-A2	10% FBS	4.4 mM	NaH_2_PO_4_	2.8 mM	7 days	37 °C	Phenol red-free DMEM medium + 1% (v/v) sodium pyruvate and 1% (v/v) NEAA and antibiotics	TBS
CPP-B2	10% FBS	4.4 mM	NaH_2_PO_4_	2.8 mM	14 days	37 °C	Phenol red-free DMEM medium + 1% (v/v) sodium pyruvate and 1% (v/v) NEAA and antibiotics	TBS
CPP-C2	1 mg/ml Bovine fetuin-A	6 mM	NaH_2_PO_4_	10 mM	12 h	37 °C	140 mM NaCl, 50 mM Tris–HCl, pH 7.4	TBS
CPP-D2	40% FBS	6 mM	NaH_2_PO_4_	10 mM	12 h	37 °C	140 mM NaCl, 50 mM Tris–HCl, pH 7.4	TBS

*NEAA* non-essential amino acids

### Transmission Electron Microscopy, Energy-Dispersive Spectroscopy and X-Ray Powder Diffraction of Synthetic CPP

For transmission electron microscopy (TEM) imaging, CPP pellets were washed in milli-Q water and transferred onto a Formvar-coated copper grid. After air-drying, high-resolution images were obtained using a JEOL JEM 1400 microscope (JEOL USA Inc., Peabody, Massachusetts, USA) with an accelerating voltage of 60 kV. Images were acquired using a Gatan Orius digital camera system (Gatan Inc., Pleasanton, California, USA). For energy-dispersive X-ray (EDX) analysis of CPP2, CPP pellets were washed in milli-Q water and airdried on copper tape for analysis. Micro-elemental analysis was performed in combination with a GeminiSEM Sigma 300 electron microscope (Zeiss, Oberkochen, Germany), using a Quantax 200 detector (Bruker, Massachusetts, USA). Measurements were obtained at an accelerating voltage of 20 kV. Elemental analysis of CPP1 were performed with an EDX arm on the JEOL JEM 1400 microscope. For X-ray diffraction (XRD) analysis, diffractograms were measured on a Panalytical Empyrean (Malvern Panalytical, Malvern, UK) in transmission mode with fine-focus sealed tube, focusing mirror and PIXcel3D detector, using CuKα radiation. The samples were measured in 0.5 mm soda glass capillaries with a wall thickness of 0.01 mm.

### Nanoparticle Tracking Analysis

CPP quantification and size distribution was analyzed by nanoparticle tracking analysis (NTA) using a NanoSight NS300, with a 488 nm laser and sCMOS camera (Malvern Panalytical, Malvern, UK). CPP pellets originating from equal volumes of CPP mixture were washed, diluted and measured in TBS. Samples were analyzed under a constant flow rate (50) at 25 °C. For each sample, three 30 s videos were captured with a camera level of 14. Data were analyzed using NTA 3.2.16 software with a detection threshold of 5.

### Ca^2+^ Measurements in CPP Samples

Ca^2+^ concentration of the CPP pellets and concentrates were dissolved in 0.1 M HCl and measured using the *o*-cresolphthalein complexone method [32]. Volumes were kept equal between the CPP samples and resulting values were corrected for the concentration factor.

### Isolation of Endogenous CPP

We created three pools of uraemic serum using samples obtained from patients undergoing chronic haemodialysis therapy for ESKD (Department of Nephrology, The Royal Melbourne Hospital), enrolled in the FLKESI prospective observational study, as previously described [27]. All participants gave written informed consent, and the study was approved by local ethics committee (Melbourne Health Research and Ethics Committee ref.: 2012.141) and was conducted in accordance with the Declaration of Helsinki. Each pool was derived from 10 unique patients using equal volumes of fresh (unfrozen) serum (10 mL) from each participant. Endogenous CPP were isolated by differential centrifugation according to published methods [5]. CPP pellets were washed twice in TBS (50 mM Tris, 140 mM NaCl, pH 7.4) and resuspended in the same buffer (1 mL) prior to estimation of particle concentrations using NTA (see below). CPP were diluted to the desired concentration using TBS and stored at 4 °C without freezing.

### Cell Culture

hVSMC were purchased from ATCC (#PCS-100-012, Manassas, Virginia, USA) and grown in medium consisting of DMEM (Lonza, Basel, Switzerland) supplemented with 20% (v/v) FBS, 2 mmol/L l-glutamine, 0.1 mmol/L non-essential amino acids and antibiotics for all experiments except for the experiment of Fig. 5G. Here, phenol red free-medium 199 (M199, Gibco) supplemented with 10% (v/v) FBS, 2 mmol/L l-glutamine and antibiotics was used and all cells were incubated at 37 °C in a humidified incubator containing 5% CO_2_ (v/v). Cells were used for experiments up to passage ten. For calcification experiments, cells were seeded in 12-well plates and grown to confluence. Experimental medium consisted of DMEM supplemented with 5% (v/v) FBS, 4 mmol/L l-glutamine, 0.1 mmol/L non-essential amino acids and antibiotics. Only 5% (v/v) FBS was used in experiments to be able to induce calcification, but not increase cytotoxicity of the VSMC during incubation. CPP2 were added at a fixed concentration according to their Ca^2+^ content equal to 100 µg Ca^2+^ per mL medium (100 CPP2) for 24 h. For the experiments based on particle number, CPP2 were added at 5 × 10^9^ particles per mL cell culture medium for 24 h. This number was based on the amount of CPP-B used when treating the cells with 100 CPP2. Additionally, CPP1 and CPP2 were added to the experimental medium in a concentration of 10^8^ particles per mL for 72 h to compare the effects to endogenous CPP, which could only be tested at lower concentrations due to their levels in vivo. For the experiment using endogenous CPP hVSMC were seeded at 2 × 10^4^ cells/cm^2^ in 24-well plates (Corning) and grown to 80% confluence. Cells were then serum-starved for 12 h in M199 with 0.5% BSA (Sigma), before switching to fresh M199 medium containing 5% EV-depleted FBS and antibiotics supplemented with 10^8^/mL CPP (endogenous or synthetic) or vehicle (TBS) 72 h.

### Analysis of VSMC Calcification

For quantification of total calcium deposition, cells were washed with phosphate-buffered saline (PBS) and decalcified in 0.1 M HCl at room temperature. Calcium concentration in the supernatant was measured using the *o*-cresolphthalein complexone method. Next, total cell lysis and total protein isolation of the cell monolayer was achieved by adding 0.1 M NaOH/0.1% (w/v) sodium dodecyl sulfate (SDS). Calcium concentration was normalized for total protein, as measured by Pierce BCA protein detection kit according to the manufacturer’s protocol (Life Technologies, Thermo Fisher Scientific). For the experiment with endogenous CPP mineralization was measured as previously described [30]. The monolayer was gently washed with 0.9% saline and the cells lysed with 0.1% SDS in 0.1 M NaOH (Sigma). Insoluble precipitates were dissolved overnight at 4 °C with 0.6 M HCl (Sigma) and the calcium concentration measured using a fluorometric probe (*Ex/Em* = 500/530 nm; #K409-100; BioVision) using a multimode plate reader (Sigma). Limit of detection 0.05 µM. Total protein content was determined using the micro-BCA assay (Pierce Scientific) and used for normalization.

### Alizarin Red Staining

For visualization of calcium deposition, alizarin red was used as previously described [33]. Briefly, cell cultures were washed with PBS and fixed in 4% (v/v) buffered formaldehyde for 15 min, washed twice with milli-Q water and stained in 2% (w/v) alizarin red (Sigma) for 5 min. Before imaging the cells were washed with milli-Q water to remove excess Alizarin Red staining.

### Statistical Analysis

Statistical analyses were conducted in GraphPad Prism 7 (San Diego, California, USA). Parametric data as identified by the Shapiro–Wilk test was analyzed by a one-way ANOVA followed by the Tukey or šídák post hoc test to correct for multiple comparisons. A *P*-value of < 0.05 was considered statistically significant. Data are presented as mean ± standard error of the mean (SE) of at least three independent experiments each consisting of at least three replicates.

## Results

### Protocol Selection

**Fig. 1:**
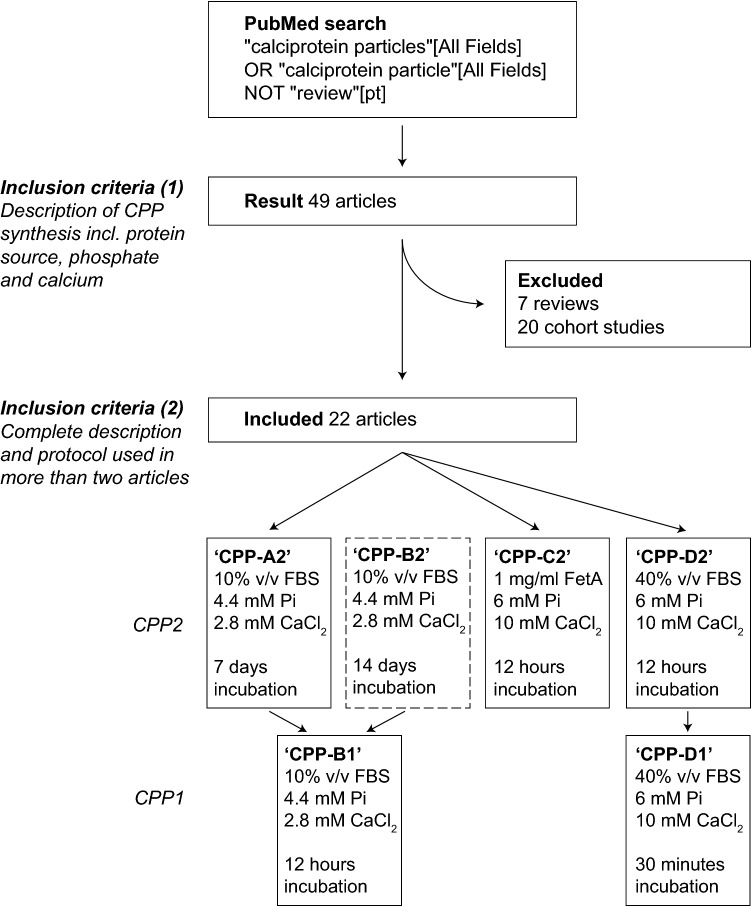
Flowchart of literature search and protocol selection. A PubMed search was performed in May 2019 which resulted in 49 articles. Twenty-seven articles were excluded because they did not synthesize CPP in vitro. The twenty-two included articles were screened and the three most used protocols were selected (Protocol A, C and D). Protocol B was added to the comparison to have more matured CPP-A2. Additionally, protocols for CPP1 A/B and D are also included

A PubMed search (“calciprotein particles” [All Fields] OR “calciprotein particle” [All Fields] NOT “review” [pt]) was performed to identify articles that describe CPP2 synthesis in the context of CKD (inclusion criteria 1). Only studies describing synthesis methods of CPP were included, resulting in exclusion of 27 out of the 49 articles (*n* = 7 review articles, *n* = 20 cohort studies). The 22 remaining articles that provided sufficient methodological details (inclusion criteria 2) are summarized in Table 1. We identified four protocols used across multiple articles and selected these for side-by-side comparison: CPP-A2, CPP-B2, CPP-C2 and CPP-D2 (Fig. 1). The protocols differ in concentrations of Ca^2+^ and P_i_, protein source and incubation time (Table 1). CPP-A2 are formed in medium containing lower amount of serum and high concentrations of Ca^2+^ and P_i_ for 7 days. Additionally, this protocol was tested with a prolonged incubation time of 14 days because this yields more ripened CPP2 (protocol CPP-B2). In CPP-C2 and CPP-D2 high levels of both Ca^2+^ and P_i_ were added to a physiological buffer of 140 mM NaCl and 50 mM Tris (pH 7.4), containing either bovine fetuin-A (CPP-C2) or 40% FBS (CPP-D2) as the protein source, requiring only an incubation time of 12 h. Additionally, two protocols to synthesize CPP1 (CPP-B1 and CPP-D1) were also considered. These protocols are identical to CPP-A/B2 and CPP-D2, with a shortened incubation time of 12 h and 30 min, respectively. CPP-C1 was too unstable in our hands, and therefore not considered further in this study.

### Characterization of CPP2 Morphology and Elemental Composition

**Fig. 2:**
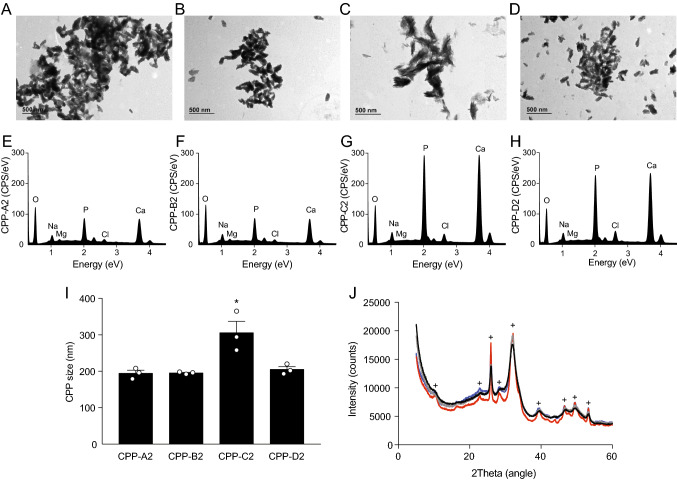
Morphological and elemental characteristics of the different CPP2. Transmission electron microscopy (TEM) images of **A** CPP-A2, **B** CPP-B2, **C** CPP-C2, **D** CPP-D2 based protocols. Scale bars correspond to 500 nm. **E**–**H** Relative amounts of electrolytes incorporated in the CPP were measured with energy-dispersive X-ray spectroscopy (EDX). **I** Size of the CPP as quantified on the TEM images. **J** With X-ray diffraction the crystallinity was determined in the differently synthesized CPP. “+” signs indicate hydroxyapatite peaks. Lines indicate CPP-A2 (grey), CPP-B2 (black), CPP-C2 (red) and CPP-D2 (blue). Data are presented as mean ± SE of three independent experiments, *P < 0.05 versus all other CPP

The different CPP2 were compared based on morphology, elemental composition and crystallinity using TEM and EDX analysis. Optically the morphology of all CPP2 was similar (Fig. 2A–D). EDX analysis demonstrated that CPP-C2 and CPP-D2 contain most P_i_ and Ca^2+^ (Fig. 2E–H). Moreover, CPP-C2 were significantly larger in long axis diameter (310 ± 20 versus 190 ± 10, 200 ± 10 and 200 ± 10 nm for CPP-A2, B2 and D2, respectively, Fig. 2I). X-ray diffraction (XRD) analysis showed that CPP-C2 contain the most crystalline hydroxyapatite compared to the other CPP2 preparations (Fig. 2J).

### Calcification Potency of CPP2

**Fig. 3:**
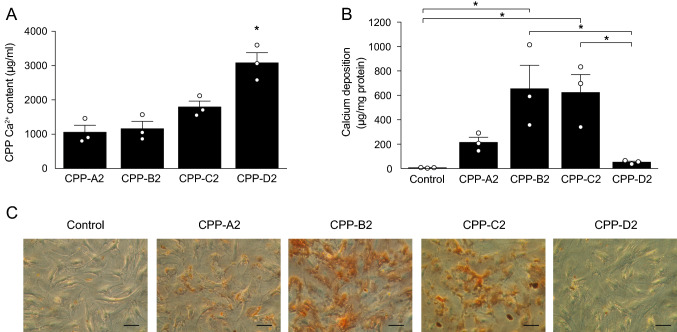
Calcium deposition after standardization by calcium content. **A** Ca^2+^ content measured in the concentrated samples of CPP with the o-cresolphthalein complexone method. VSMC were incubated with CPP volumes equal to 100 μg Ca/ml. **B** Ca^2+^ deposition was measured after 24 h and **C** alizarin red staining was performed to visualize calcification. Scale bars correspond to 100 μm. Data are presented as mean ± SE of three independent experiments, *P < 0.05

To test calcification potency of the different CPP2 preparations, we treated hVSMC for 24 h with a fixed amount of CPP2 spiked into the culture medium. In the first set of experiments, the Ca^2+^ content of the CPP2 was used to standardize the amount added; here we used 100 µg Ca^2+^ per ml medium (100 CPP2), as this has been widely used in other in vitro studies of CPP [14–16, 26, 30]. The Ca^2+^ content of CPP-D2 (3080 ± 290 µg/mL Ca^2+^) was significantly higher than CPP-A2 (1060 ± 210 µg/mL Ca^2+^), CPP-B2 (1160 ± 210 µg/mL Ca^2+^) and CPP-C2 (1800 ± 170 µg/mL Ca^2+^, Fig. 3A). Supplementation of CPP2 standardized to the amount of Ca^2+^ content led to variable calcium deposition (Fig. 3B), in which CPP-D2 induced least calcification (50 ± 10 µg Ca^2+^/mg protein). Stimulation with CPP-A2 resulted in less calcification (210 ± 40 µg Ca^2+^/mg protein) compared to CPP-B2 (650 ± 190 µg Ca^2+^/mg protein) and CPP-C2 (620 ± 150 µg Ca^2+^/mg protein). The highest calcification potency of CPP-B2 and CPP-C2 was confirmed visually with alizarin red staining of hVSMC (Fig. 3C).

**Fig. 4:**
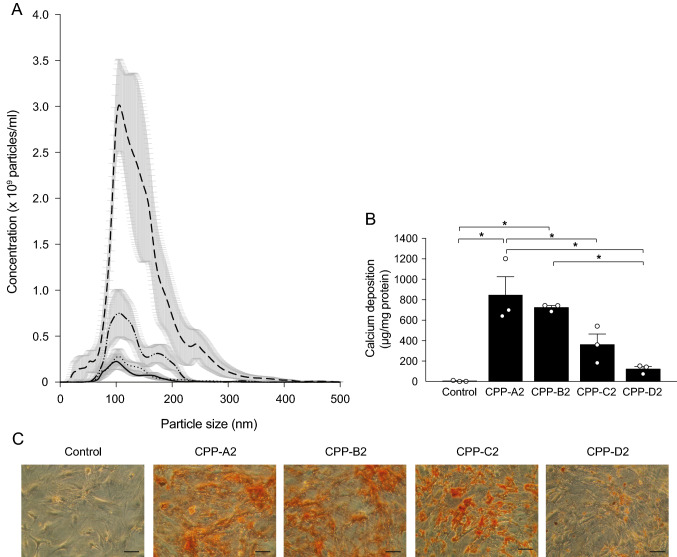
Calcium deposition after standardization by CPP2 particle number. **A** Distribution of the number and size of particles measured by nanoparticle tracking analysis (NTA) of CPP-A2 (solid line), CPP-B2 (dotted line), CPP-C2 (intermittent line), CPP-D2 (intermittent dotted line) protocols. **B** Calcification deposition of the CPP was studied after standardizing for particles quantity using and hVSMC were incubated with 5 × 10^9^ particles/ml and **C** alizarin red staining was performed to visualize calcification. Scale bars correspond to 100 μm. Data are presented as mean ± SE of three independent experiments, *P < 0.05

However, because the Ca^2+^ content does not necessarily correspond with the number of particles present, we repeated the hVSMC experiment keeping the number of CPP2 constant. CPP2 quantity was measured using NTA (Fig. 4A) and expressed as the particle number per ml. Interestingly, the distribution of CPP2 particle numbers showed a different pattern to the measured Ca^2+^ content. CPP-C2 yielded the highest number of particles (2.4 × 10^11^ ± 8.3 × 10^10^) compared to the other protocols (1.4 × 10^10^ ± 2.1 × 10^9^ (CPP-A2), 3.6 × 10^10^ ± 1.4 × 10^9^ (CPP-B2) and 7.1 × 10^10^ ± 1.2 × 10^10^ particles/ml (CPP-D2), Fig. 4A). After treatment of hVSMC with 5 × 10^9^ particles per ml medium, which approximates to the particle number in 100 CPP-B2, calcification measurements were similar between CPP-A2 and CPP-B2 (850 ± 180 versus 720 ± 20 µg Ca^2+^/mg protein, Fig. 4B). Compared to CPP-A2, calcium deposition was significantly lower in CPP-C2 (850 ± 180 versus 360 ± 100 µg Ca^2+^/mg protein). CPP-D2 was least potent to induce hVSMC calcification (120 ± 20 µg Ca^2+^/mg protein), which was confirmed by Alizarin Red S staining in hVSMC (Fig. 4C).

Another option to normalize the quantity CPP to, is using total protein concentration. However, the protein content shows the same trend as found with calcium content between the protocols (Supplemental Fig. S1). CPP-D2 had the highest protein content (3.7 ± 0.1 mg/ml), second most CPP-C2 (1.4 ± 0.1 mg/ml) and CPP-A2 and CPP-B2 show the same lowest protein content (0.8 ± 0.1 and 0.9 ± 0.1 mg/ml, respectively). Normalization to protein content would therefore not change the calcification potency of the different CPP2 preparations.

### Comparison Between Endogenous and Synthetically Made CPP

**Fig. 5:**
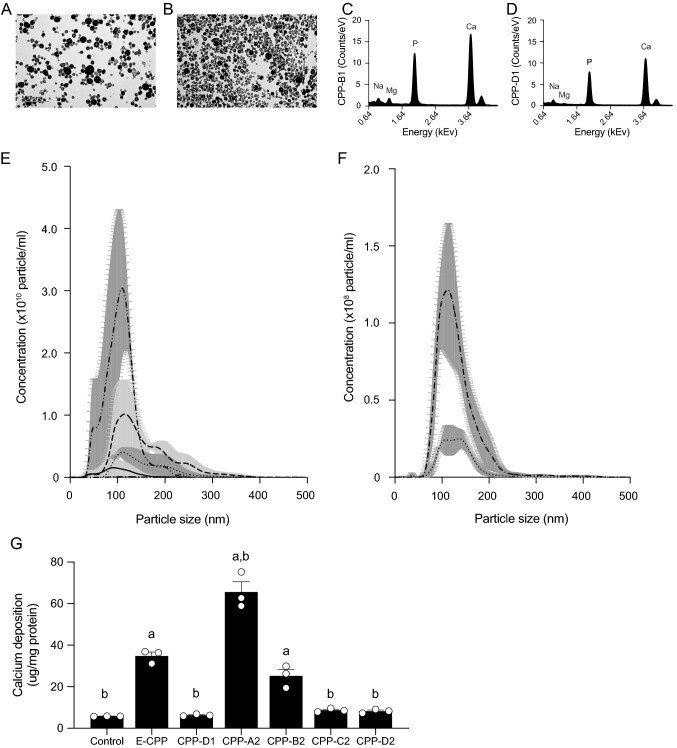
Calcium deposition after stimulation with physiological levels of endogenous CPP, CPP1 and CPP2. Transmission electron microscopy (TEM) images of **A** CPP-B1, **B** CPP-D. Relative amounts of electrolytes incorporated in the CPP were measured with energy-dispersive X-ray spectroscopy for **C** CPP-B1 and **D** CPP-D1. **E** Distribution of the number and size of particles measured by nanoparticle tracking analysis (NTA) of CPP-A2 (solid line), CPP-B2 (dotted line), CPP-C2 (intermittent dashed line), CPP-D2 (intermittent dashed dotted line) protocols. **F** NTA analysis of CPP-B1 (dotted line) and CPP-D1 (intermittent line). **G** Calcification deposition of the CPP was studied after standardizing for particle number and hVSMC were incubated with 10^8^ particles/ml. Data are presented as mean ± SE of three independent experiments. Significance (*p* < 0.001) is depicted as different from control (**a**) or E-CPP (**b**). E-CPP, endogenous CPP

To determine which protocol of in vitro generated CPP that best resembles native CPP, we isolated CPP from the pooled serum of patients requiring haemodialysis therapy. In this endogenous CPP sample, CPP1 and CPP2 were identified by their characteristic morphology on cryo-TEM, with CPP2 only representing 8% of the particles observed. Semiquantitative XRD analysis identified these CPP samples as mostly amorphous in addition to a minor crystalline component, consistent with the predominance of CPP1 (Supplemental Fig. S2). NTA analysis revealed particle numbers equivalent to 1.1 × 10^7^ ± 7.8 × 10^6^ particles/ml in serum (Supplemental Fig. S3), which is several orders of magnitude lower than the amount of 5 × 10^9^ particles/ml that was used in this study and elsewhere. Consequently, we adapted the experimental conditions of our in vitro experiments to match the levels of endogenous CPP. First, we generated CPP-B1 and CPP-D1 in addition to the previously tested CPP2 to assess the comparative calcification potency of CPP1. TEM pictures of CPP-B1 and CPP-D1 confirmed successful synthesis of CPP1 (Fig. 5A, B). Elemental analysis using EDX confirmed high calcium and phosphorus levels in the CPP1 (Fig. 5C, D). Second, we used a lower number of particles/ml (10^8^) and prolonged hVSMC stimulation time of 72 h to compare calcification potency of CPP1 and CPP2 to endogenous CPP. No increased calcium deposition was measured after hVSMC exposed to CPP-D1 (6 ± 0), CPP-C2 (9 ± 1) and CPP-D2 (8 ± 1) compared to control (6 ± 0 µg Ca^2+^/mg protein, Fig. 5G). Likewise, CPP-B1 did not increase calcification (8 ± 6, Supplemental Fig. S4A). Endogenous CPP (E-CPP), CPP-A2 and CPP-B2 significantly increased calcium deposition compared to control (E-CPP (35 ± 2), CPP-A2 (66 ± 5), CPP-B2 (25 ± 3 µg Ca^2+^/mg protein, Fig. 5G). Calcification of CPP-B2 was comparable to endogenous CPP calcification, whereas CPP-A2 was more potent to calcify than endogenous CPP.

### Stability of CPP2 After Prolonged Storage

To assess the stability of CPP2, calcification potency was studied after prolonged storage at 4 °C or following a freeze–thaw cycle after storage at – 80 °C. After 14 days of storage, Ca^2+^ content was measured again and all Ca^2+^ contents were comparable to the freshly measured Ca^2+^ contents (Supplemental Fig. S5A). Additionally, the corresponding calcium deposition in hVSMC cultures was not significantly different from the calcification measured with fresh CPP2 samples across all four CPP2 types (Supplemental Fig. S5B).

## Discussion

In this study, we show that commonly used protocols for CPP synthesis result in particles of different sizes, composition and in vitro calcification potency. Our systematic comparison of four widely used protocols to generate CPP, demonstrate that (1) the incubation with fetuin-A compared to FBS results in larger particles, (2) the use of high Ca^2+^ and P_i_ concentration increases the Ca^2+^ content and (3) the incubation based on Ca^2+^ content normalization results in significant differences in particle numbers. Altogether, these findings demonstrate the heterogeneity of experimental conditions applied in CPP research that greatly hampers reliable comparisons between the various studies.

Our most striking finding is that the Ca^2+^ content of CPP is not a reliable method to quantify CPP particles, when comparing different synthetic protocols. Measuring Ca^2+^ content, is a fast, cheap and the most used method to quantify the amount of CPP whereas determining the number of particles by nanoparticle tracking analysis (NTA) is more time-consuming, more expensive and less widely used. Indeed, even in the minority of studies where NTA was performed [5, 10, 14], Ca^2+^ content was still used to determine treatment dose. Our data clearly demonstrate that these two methods of quantitation do not correlate. Perhaps more importantly, the Ca^2+^ content of CPP is not predictive of the calcification potency in vitro. In this study, CPP-D2 demonstrated the highest Ca^2+^ content of all CPP mixtures. However, CPP-D2 was not potent to calcify VSMC in all conditions. CPP-D2 also contained the highest total protein content, which could inhibit its calcification potency. Based on our analysis, this experimental approach should be reconsidered to enable better comparison between studies.

To our knowledge, we are the first to compare a large number of different protocols to synthesize both CPP1 and CPP2 and investigate their calcification potency on hVSMC. Previous studies have shown the calcification potency of CPP-A2 and CPP-B2 [14–16] and CPP-D2 [30], but protocol CPP-C2 was never tested on its calcification potency. As CPP maybe key drivers of calcification, most protocols are used in vitro to investigate different aspects of the calcification process, including VSMC calcification.

Previously, a few studies compared endogenous CPP and synthetic CPP [5, 34]. Calcium phosphate particles isolated from atherosclerotic plagues and synthetic counterparts were compared on morphology, chemical properties and endothelial toxicity [34], concluding that there are no distinct differences. These natural and synthetic particles were cultured using supplementation of 1 mmol/L CaCl_2_ and 1 mmol/L Na_2_HPO_4_ in DMEM containing 10% (v/v) FBS and incubated for 6 weeks, which is a similar, but longer, protocol as the CPP-A/B2. Despite the very long maturation time, these synthetic particles did not calcify bovine and porcine pericardium, but induced apoptosis-mediated endothelial toxicity. Likewise, protein content of endogenous CPP compared to uremic human serum derived CPP2 (comparable to CPP-D2) reflected the same predicted biological effects in pathways such as atherosclerosis, coagulation and complement system [5].

Endogenous CPP from CKD patients are a mixture of CPP1 and CPP2 [5]. Therefore, both CPP1 and CPP2 were tested in our experimental set-up. In contrast to CPP2, CPP1 are not able to induce calcification in VSMC, confirming previous findings [14]. As shown here in this study and by others, CPP1 consist of amorphous material and are spherical entities, whereas CPP2 resemble a crystalline structure and are ellipsoid shaped particles [5, 9]. Probably this crystalline structure explains the increased potency of CPP2 to calcify tissues compared to CPP1. In healthy individuals, calciprotein monomers (CPM) may be the predominant form of protein-mineral complexes in the circulation [10, 35]. This very small (± 9 nm in diameter) particle can cross the glomerular filtration barrier and is cleared by proximal tubule cells [35], whereas the larger CPP1 and CPP2 are mainly cleared by the liver and spleen [21, 24, 36]. Physiologically these particles are interesting because they do not only circulate in CKD patients, but also in the general population. CPM were in different cell types not cytotoxic and were not able to induce an inflammatory response, in contrast to CPP1 and CPP2 [35]. It has been suggested that CPM play a role in the negative feedback loop to keep serum phosphate levels within the normal range, rather than inducing ectopic calcification [36].

Our data demonstrates that endogenous CPP are able to induce low-level VSMC calcification with particle numbers equivalent to those observed in some dialysis patients with poorly controlled mineral balance [37]. Endogenous CPP contain more fetuin-A compared to synthetic CPP [5] and have a less crystalline structure [8]. Multiple studies showed that fetuin-A is an inhibitor of soft-tissue calcification [8, 38–40]. Fetuin-A stabilizes the amorphous phase after Ca-Pi aggregation and delays crystallization into hydroxyapatite [9, 25]. Once matured into CPP2, the fetuin-A coat shields the crystalline core to prevent further growth and mediates safe disposal via macrophages [21, 24, 41]. Nevertheless, CPP-C2 that were formed solely using fetuin-A as a protein source were largest and most crystalline in our experiments.

The observation that fetuin-A enables most efficient CPP2 formation and potently induces VSMC calcification is of interest. A potential explanation is that, in addition to fetuin-A, other serum proteins determine CPP stabilization and their cytotoxicity [6, 42]. These proteins were absent in the synthesis protocol of CPP-C2, but are present in endogenous CPP [5, 12]. However, supplementation of fetuin-A to aqueous solutions containing Ca^2+^ and P_i_ prevented Ca-Pi crystallization effectively [43]. In vivo fetuin-A likely acquire other serum proteins from serum to the CPP [44]. Therefore, the translational value of CPP synthesized in presence of fetuin-A but in absence of other serum proteins is questionable, as endogenous CPP never solely contain fetuin-A. The differences in protein composition may therefore partly explain difference in the calcification potency of CPP2.

Formation of endogenous CPP is dependent on serum proteins available in the individual patient. In CKD patients the levels of fetuin-A and other proteins are lower than in healthy adults [7]. Moreover, CPP formed in uremic serum have been demonstrated to be uniquely enriched for carbonate-substituted apatite, DNA fragments, small RNA and microbe-derived components [5]. Recently, it was shown that after feeding CPM, CPP1 and CPP2 are formed, probably as a buffering system to handle high loads of calcium and phosphate. This was found to form in both healthy adults and in CKD patients stage 3 or higher, with a more pronounced effect of CPM formation in CKD patients [45]. This implies that CKD patients have an impaired buffering system for calcium and phosphate loads. Additionally, CKD patients have high levels of protein bound uremic toxins such as indoxyl sulfate and *p*-cresyl sulfate [46]. Although further research is required, these factors may contribute to the higher potency of endogenous CPP to calcify VSMC.

A strength of this study it that we compared six different protocols to generate CPP1 and CPP2. Although multiple studies already showed VSMC calcification after CPP stimulation [14–16, 23, 30], we are the first to compare the effect of different synthetic CPP on VSMC calcification. Our results highlight that not all protocols yield the same CPP. The main limitation of our study is that we did not consider different sources of serum (e.g., from dialysis patients), which could affect translation of our results for CKD conditions. Moreover, our study was biased towards more frequently described CPP generation protocols. It should be noted that additional protocols have been described [16, 47–49]. The multitude of CPP generation protocols highlights the importance of our study. A standardized protocol to synthesize CPP would improve the reproducibility and the comparability of studies with synthetic CPP.

To conclude, this study demonstrates that it is important to standardize CPP synthesis protocols. Based on our results, we recommend the use of serum instead of fetuin-A alone as a source of protein, lower CPP concentrations determined by particle number, and to consider CPP1 in the experimental set-up to better mimic the in vivo environment of CKD patients.

## Supplementary Information

Below is the link to the electronic supplementary material.

Supplementary file1 (DOCX 17517 KB)

## References

[1] Blacher J, Guerin AP, Pannier B (2001). Arterial calcifications, arterial stiffness, and cardiovascular risk in end-stage renal disease. Hypertension.

[2] Block GGA, Hulbert-Shearon TE, Levin NWN, Port FFK (1998). Association of serum phosphorus and calcium x phosphate product with mortality risk in chronic hemodialysis patients: a national study. Am J Kidney Dis.

[3] Block GA, Klassen PS, Lazarus JM (2004). Mineral metabolism, mortality, and morbidity in maintenance hemodialysis. J Am Soc Nephrol.

[4] Eddington H, Hoefield R, Sinha S (2010). Serum phosphate and mortality in patients with chronic kidney disease. Clin J Am Soc Nephrol.

[5] Smith ER, Hewitson TD, Hanssen E, Holt SG (2018). Biochemical transformation of calciprotein particles in uraemia. Bone.

[6] Pasch A, Farese S, Gräber S (2012). Nanoparticle-based test measures overall propensity for calcification in serum. J Am Soc Nephrol.

[7] Hamano T, Matsui I, Mikami S (2010). Fetuin-mineral complex reflects extraosseous calcification stress in CKD. J Am Soc Nephrol.

[8] Heiss A, DuChesne A, Denecke B (2003). Structural basis of calcification inhibition by alpha 2-HS glycoprotein/fetuin-A. Formation of colloidal calciprotein particles. J Biol Chem.

[9] Heiss A, Eckert T, Aretz A (2008). Hierarchical role of fetuin-A and acidic serum proteins in the formation and stabilization of calcium phosphate particles. J Biol Chem.

[10] Miura Y, Iwazu Y, Shiizaki K (2018). Identification and quantification of plasma calciprotein particles with distinct physical properties in patients with chronic kidney disease. Sci Rep.

[11] Smith ER, Ford ML, Tomlinson LA (2014). Serum calcification propensity predicts all-cause mortality in predialysis CKD. J Am Soc Nephrol.

[12] Viegas CSBB, Santos L, Macedo AL (2018). Chronic kidney disease circulating calciprotein particles and extracellular vesicles promote vascular calcification. Arterioscler Thromb Vasc Biol.

[13] Smith ER, Ford ML, Tomlinson LA (2012). Phosphorylated fetuin-A-containing calciprotein particles are associated with aortic stiffness and a procalcific milieu in patients with pre-dialysis CKD. Nephrol Dial Transplant.

[14] Aghagolzadeh P, Bachtler M, Bijarnia R (2016). Calcification of vascular smooth muscle cells is induced by secondary calciprotein particles and enhanced by tumor necrosis factor-α. Atherosclerosis.

[15] Aghagolzadeh P, Radpour R, Bachtler M (2017). Hydrogen sulfide attenuates calcification of vascular smooth muscle cells via KEAP1/NRF2/NQO1 activation. Atherosclerosis.

[16] Ter Braake AD, Eelderink C, Zeper LW (2020). Calciprotein particle inhibition explains magnesium-mediated protection against vascular calcification. Nephrol Dial Transplant.

[17] Kuro-o M (2014). Calciprotein particle (CPP): a true culprit of phosphorus woes?. Nefrologia.

[18] Chen W, Anokhina V, Dieudonne G (2019). Patients with advanced chronic kidney disease and vascular calcification have a large hydrodynamic radius of secondary calciprotein particles. Nephrol Dial Transplant.

[19] Heiss A, Jahnen-Dechent W, Endo H, Schwahn D (2007). Structural dynamics of a colloidal protein-mineral complex bestowing on calcium phosphate a high solubility in biological fluids. Biointerphases.

[20] Heiss A, Pipich V, Jahnen-Dechent W, Schwahn D (2010). Fetuin-A is a mineral carrier protein: small angle neutron scattering provides new insight on Fetuin-A controlled calcification inhibition. Biophys J.

[21] Herrmann MM, Schäfer C, Heiss A (2012). Clearance of fetuin-A-containing calciprotein particles is mediated by scavenger receptor-A. Circ Res.

[22] Ismail AH, Schäfer C, Heiss A (2011). An electrochemical impedance spectroscopy (EIS) assay measuring the calcification inhibition capacity in biological fluids. Biosens Bioelectron.

[23] Kelynack KJ, Holt SG, Hewitson TD, Smith ER, Holt SG (2016). An in vitro murine model of vascular smooth muscle cell mineralization. Kidney research methods in molecular biology.

[24] Köppert S, Büscher A, Babler A (2018). Cellular clearance and biological activity of calciprotein particles depend on their maturation state and crystallinity. Front Immunol.

[25] Rochette CN, Rosenfeldt S, Heiss A (2009). A shielding topology stabilizes the early stage protein-mineral complexes of Fetuin-A and calcium phosphate: a time-resolved small-angle x-ray study. ChemBioChem.

[26] Smith ER, Hanssen E, McMahon LP, Holt SG (2013). Fetuin-A-containing calciprotein particles reduce mineral stress in the macrophage. PLoS ONE.

[27] Smith ER, Hewitson TD, Cai MMX (2017). A novel fluorescent probe-based flow cytometric assay for mineral-containing nanoparticles in serum. Sci Rep.

[28] Wu CY, Young D, Martel J, Young JD (2015). A story told by a single nanoparticle in the body fluid: demonstration of dissolution-reprecipitation of nanocrystals in a biological system. Nanomedicine.

[29] Cai MM, Wigg B, Smith ER (2015). Relative abundance of fetuin-A in peritoneal dialysis effluent and its association with in situ formation of calciprotein particles: an observational pilot study. Nephrology.

[30] Cai MMX, Smith ER, Tan S-J (2017). The role of secondary calciprotein particles in the mineralisation paradox of chronic kidney disease. Calcif Tissue Int.

[31] Chabrière E, Gonzalez D, Azza S (2014). Fetuin is the key for nanon self-propagation. Microb Pathog.

[32] Gitelman J (1967). An improved automated procedure of calcium in biological for the determination specimens. Anal Biochem.

[33] ter Braake AD, Tinnemans PT, Shanahan CM (2018). Magnesium prevents vascular calcification in vitro by inhibition of hydroxyapatite crystal formation. Sci Rep.

[34] Kutikhin AG, Velikanova EA, Mukhamadiyarov RA (2016). Apoptosis-mediated endothelial toxicity but not direct calcification or functional changes in anti-calcification proteins defines pathogenic effects of calcium phosphate bions. Sci Rep.

[35] Koppert S, Ghallab A, Peglow S (2021). Live imaging of calciprotein particle clearance and receptor mediated uptake: role of calciprotein monomers. Front Cell Dev Biol.

[36] Akiyama K, Miura Y, Hayashi H (2020). Calciprotein particles regulate fibroblast growth factor-23 expression in osteoblasts. Kidney Int.

[37] Ruderman I, Smith ER, Toussaint ND (2018). Longitudinal changes in bone and mineral metabolism after cessation of cinacalcet in dialysis patients with secondary hyperparathyroidism. BMC Nephrol.

[38] Reynolds JL, Skepper JN, McNair R (2005). Multifunctional roles for serum protein fetuin-A in inhibition of human vascular smooth muscle cell calcification. J Am Soc Nephrol.

[39] Schäfer C, Heiss A, Schwarz A (2003). The serum protein α2–Heremans-Schmid glycoprotein/fetuin-A is a systemically acting inhibitor of ectopic calcification. J Clin Invest.

[40] Jahnen-Dechent W, Heiss A, Schäfer C, Ketteler M (2011). Fetuin-A regulation of calcified matrix metabolism. Circ Res.

[41] Cai MM, Smith ER, Holt SG (2015). The role of fetuin-A in mineral trafficking and deposition. Bonekey Rep.

[42] Dautova Y, Kozlova D, Skepper JN (2014). Fetuin-A and albumin alter cytotoxic effects of calcium phosphate nanoparticles on human vascular smooth muscle cells. PLoS ONE.

[43] Price PA, Lim JE (2003). The inhibition of calcium phosphate precipitation by fetuin is accompanied by the formation of a fetuin-mineral complex. J Biol Chem.

[44] Tenzer S, Docter D, Kuharev J (2013). Rapid formation of plasma protein corona critically affects nanoparticle pathophysiology. Nat Nanotechnol.

[45] Tiong MK, Cai MMX, Toussaint ND (2022). Effect of nutritional calcium and phosphate loading on calciprotein particle kinetics in adults with normal and impaired kidney function. Sci Rep.

[46] Tiong MK, Krishnasamy R, Smith ER (2021). Effect of a medium cut-off dialyzer on protein-bound uremic toxins and mineral metabolism markers in patients on hemodialysis. Hemodial Int.

[47] Shishkova DK, Velikanova EA, Bogdanov LA (2021). Calciprotein particles link disturbed mineral homeostasis with cardiovascular disease by causing endothelial dysfunction and vascular inflammation. Int J Mol Sci.

[48] Murthy S, Karkossa I, Schmidt C (2022). Danger signal extracellular calcium initiates differentiation of monocytes into SPP1/osteopontin-producing macrophages. Cell Death Dis.

[49] Anzai F, Karasawa T, Komada T (2021). Calciprotein particles induce IL-1β/α–mediated inflammation through NLRP3 inflammasome-dependent and -independent mechanisms. ImmunoHorizons.

