# Supplementary material for: Calciprotein Particle Synthesis Strategy Determines In Vitro Calcification Potential
Source: Calcif Tissue Int. 2022 Nov 3;112(1):103–17. doi: 10.1007/s00223-022-01036-1 (PMC9813048; doi:10.1007/s00223-022-01036-1)
Supplement: Supplementary file 1 — Supplementary file1 (DOCX 17517 KB) [file 223_2022_1036_MOESM1_ESM.docx]

**SUPPLEMENTAL DATA AND METHODS**

**Calciprotein particle synthesis strategy determines *in vitro* calcification potential**

Lara W. Zeper^1^, Edward R. Smith^2,3^, Anique D. ter Braake^1^, Paul T. Tinnemans^4^, Jeroen H.F. de Baaij^1^, Joost G.J. Hoenderop^1^

^1^Department of Physiology, Radboud Institute for Molecular Life Sciences, Radboud University Medical Center, Nijmegen, The Netherlands

^2^Department of Nephrology, The Royal Melbourne Hospital^.^

^3^Department of Nephrology, University of Melbourne, Parkville, Victoria, Australia.

^4^Institute for Molecules and Materials, Radboud University, Nijmegen, The Netherlands

Corresponding author:

Prof. dr. Joost G.J. Hoenderop, Department of Physiology, Radboud Institute for Molecular Life Sciences, Radboud university medical center, P.O. Box 9101, 6500HB, Nijmegen, The Netherlands. Phone: (+31) 24 3617347, E-mail: [joost.hoenderop@radboudumc.nl](mailto:joost.hoenderop@radboudumc.nl)

**SUPPLEMENTAL FIGURES AND LEGENDS**

**Supplemental Figure S1 – Calcium content plotted versus total protein content.** Calcium content is plotted as black bars with white dots and correspond to the left Y-axis. Total protein content is plotted as white bars with black triangles and correspond to the right Y-axis. Data is presented as mean ± SE of three independent experiments***.***

**Supplemental Figure S2 – Endogenous CPP characterization.** (A) Cryogenic transmission electron microscopy (TEM) images of a pool of endogenous CPP isolated from serum of dialysis patients. Arrow indicates a CPP2 like structure. (B) X-ray diffraction pattern of the endogenous CPP pool. (C) Semi-quantitative estimates for identified crystalline phases, normalized to the 200 halite peak.

**Supplemental Figure S3 – Nanoparticle tracking analysis of endogenous CPP.** The endogenous pool of CPP were captured five times for 60 seconds each for nanoparticle tracking analysis. (A) Individual capture results. (B) Mean size and concentration of all five captures.

**Supplemental Figure S4– Calcium deposition after stimulation with physiological levels of CPP1 and CPP2.** (A) Calcification deposition of the CPP was studied after standardizing for particle number and hVSMC were incubated with 10^8^ particles/ml. (B) Alizarin red staining was performed to visualize calcification. Scale bars correspond to 100 μm. Data are presented as mean ± SE of four independent experiments.

**Supplemental Figure S5 – CPP calcification potency after prolonged storage.** Ca^2+^ content measured in the concentrated samples of CPP after storage at 4°C (striped bars) or -80°C (white bars) for 14 days compared to freshly measured Ca^2+^ content (black bars, A). Based on these Ca^2+^ values, VSMC were incubated with CPP volumes equal to 100 μg Ca^2+^/ml. Cellular Ca^2+^ deposition of hVSMC was quantified after 24 hours incubation (B). Data are presented as mean ± SE of three independent experiments.

**SUPPLEMENTAL MATERIALS AND METHODS**

*Protein measurements in CPP samples*

Total protein of the CPP samples were measured using the Pierce BCA protein detection kit according to the manufacturer’s protocol (Life Technologies, Thermo Fisher Scientific).

*Isolation of endogenous CPP*

We created three pools of uremic serum using samples obtained from patients undergoing chronic hemodialysis therapy for ESKD (Department of Nephrology, The Royal Melbourne Hospital), enrolled in the FLKESI prospective observational study, as previously described [1]. All participants gave written informed consent, and the study was approved by local ethics committee (Melbourne Health Research and Ethics Committee ref.: 2012.141) and was conducted in accordance with the Declaration of Helsinki. Each pool was derived from 10 unique patients using equal volumes of fresh (unfrozen) serum (10mL) from each participant. Endogenous CPP were isolated by differential centrifugation according to published methods [2]. CPP pellets were washed twice in TBS (50mM Tris, 140mM NaCl, pH 7.4) and resuspended in the same buffer (1mL) prior to estimation of particle concentrations using NTA (see below). CPP were diluted to the desired concentration using TBS and stored at 4°C without freezing.

*Cryogenic TEM endogenous CPP*

Cryogenic (cryo)- transmission electron microscopy (TEM) was performed on the endogenous CPP as previously described [3]. Briefly, particles were pelleted from diluted serum by centrifugation (30,000 g, 2h, 4°C), washed three times in TBS and re-suspended in 0.22μm filtered MilliQ water. Samples were plunged frozen in liquid ethane before observation on a Tecnai F30 (FEI, Netherlands) operating at 300kV.

*X-ray diffraction endogenous CPP*

X-ray diffraction data were collected using a Bruker D8 Advance X-ray diffractometer (Preston, Vic, Australia) with a Ni-filtered Cu Kα radiation source (1.79 Å). Data were collected between 5 – 85° 2θ, with a step size of 0.02° and a scan rate of 0.5 s per step. An anti-scatter blade was used to reduce the diffracted background intensity at low angles. An incident beam divergence of 0.26° was used with a 2.5° soller slit in the diffracted beam. The sample was spun at 15 revolutions per minute. Phase identification and semi-quantification was completed using Bruker Diffracplus EVA software with the ICDD PDF4+ 2015 database. Peaks were identified using a consistent peak width and threshold for all samples. Amorphous contribution was calculated using background subtraction in EVA with a curvature and threshold of 1.

*Nanoparticle tracking analysis endogenous CPP*

Nanoparticle tracking analysis: Nanoparticle tracking analysis (NTA) was performed on a Nanosight NS500 (Nanosight, Amesbury, UK) equipped with blue (488nm) laser, sCMOS camera and using NTA 3.2 analytical software. Samples were analysed at two dilutions (1 in 100; 1 in 1,000 in twice-0.22μm filtered TBS) to encompass the linear working range on the instrument (10^6^ to 10^9^ particles/mL), each for 5 x 60s captures, and the results averaged. Capture settings (camera level 11; slide shutter 890; slider gain: 146; frames/s: 25; syringe pump speed: 50 μL/min) and detection settings (threshold: 4; blur: auto; max jump distance: auto) were kept constant.

Cell culture

hVSMC were purchased from ATCC (#PCS-100-012, Manassas, Virginia, USA) and grown in medium consisting of DMEM (Lonza, Basel, Switzerland) supplemented with 20% (v/v) FBS, 2 mmol/L L-glutamine, 0.1 mmol/L non-essential amino acids and antibiotics or phenol red free-medium 199 (M199, Gibco) supplemented with 10% (v/v) FBS, 2 mmol/L L-glutamine and antibiotics at 37°C in a humidified incubator containing 5% CO_2_ (v/v). Cells were used for experiments up to passage ten. For calcification experiments, cells were seeded in 12-well plates and grown to confluence. Experimental medium consisted of DMEM supplemented with 5% (v/v) FBS, 4 mmol/L L-glutamine, 0.1 mmol/L non-essential amino acids and antibiotics. CPP1 and CPP2 were added to the experimental medium in a concentration of 10^8^ particles per mL for 72 hours.

Analysis of VSMC calcification

For quantification of total calcium deposition, cells were washed with phosphate-buffered saline (PBS) and decalcified in 0.1 M HCl at room temperature. Calcium concentration in the supernatant was measured using the *o*-cresolphthalein complexone method. Next, total cell lysis and total protein isolation of the cell monolayer was achieved by adding 0.1 M NaOH/0.1% (w/v) sodium dodecyl sulfate. Calcium concentration was normalized for total protein, as measured by Pierce BCA protein detection kit according to the manufacturer’s protocol (Life Technologies, Thermo Fisher Scientific).

*Alizarin Red staining*

For visualization of calcium deposition, alizarin red was used as previously described [4]. Briefly, cell cultures were washed with PBS and fixed in 4% (v/v) buffered formaldehyde for 15 minutes, washed twice with milli-Q water and stained in 2% (w/v) alizarin red (Sigma) for 5 minutes.

Statistical analysis

Statistical analyses were conducted in GraphPad Prism 7 (San Diego, California, USA). Parametric data as identified by the Shapiro-Wilk test was analyzed by a one-way ANOVA followed by the Tukey or šídák post-hoc test to correct for multiple comparisons. A *P*-value of < 0.05 was considered statistically significant. Data are presented as mean ± standard error of the mean (SE) of at least three independent experiments each consisting of at least three replicates.

**REFERENCES**

1. Smith ER, Hewitson TD, Cai MMX, et al (2017) A novel fluorescent probe-based flow cytometric assay for mineral-containing nanoparticles in serum. Sci Rep 7:5686. https://doi.org/10.1038/s41598-017-05474-y

2. Smith ER, Hewitson TD, Hanssen E, Holt SG (2018) Biochemical transformation of calciprotein particles in uraemia. Bone 110:355–367. https://doi.org/10.1016/j.bone.2018.02.023

3. Smith ER, Hanssen E, McMahon LP, Holt SG (2013) Fetuin-A-containing calciprotein particles reduce mineral stress in the macrophage. PLoS One 8:e60904. https://doi.org/10.1371/journal.pone.0060904

4. ter Braake AD, Tinnemans PT, Shanahan CM, et al (2018) Magnesium prevents vascular calcification in vitro by inhibition of hydroxyapatite crystal formation. Sci Rep 8:2069. https://doi.org/10.1038/s41598-018-20241-3
